# Graham Little-Piccardi-Lassueur syndrome with unique distribution in a man with autoimmune disease

**DOI:** 10.1016/j.jdcr.2024.07.016

**Published:** 2024-08-03

**Authors:** Katlyn M. Smaha, Victoria M. Madray, Matthew R. Powell, Loretta S. Davis

**Affiliations:** a Medical College of Georgia at Augusta University, Augusta, Georgia; b Department of Dermatology, Medical College of Georgia at Augusta University, Augusta, Georgia; c Department of Pathology, Medical College of Georgia at Augusta University, Augusta, Georgia

**Keywords:** autoimmune disease, Graham Little-Piccardi-Lassueur syndrome, lichen planopilaris

## Introduction

Graham Little-Piccardi-Lassueur syndrome (GLPLS), a rare subtype of lichen planopilaris (LPP), is classically described as a triad of fibrosing alopecia of the scalp, nonfibrosing alopecia of the axillae and groin, and follicular spinous papules over the body.^1^ The disease is often chronic, manifesting over months to years, and the complete triad is not present in all cases.

GLPLS is more common in women, with few cases reported in men.^2^, ^3^, ^4^ While its pathophysiology and predisposing factors are not well understood, it is thought to be a T-cell-mediated immunologic disorder.^5^ We report a case of atypical GLPLS in a man with involvement of the chest and lower extremities and sparing of the axillae and groin. His medical history was notable for autoimmune dysautonomia and ulcerative colitis (UC), conditions not previously reported in association with GLPLS.

## Case report

A 42-year-old man with long-standing LPP of the scalp presented with a burning, pruritic rash of his bilateral legs, worsening over several months. He associated the onset of the rash with an antecedent sunburn with no other identifiable triggers. Notably, the groin and axillae were spared. Medical history was significant for autoimmune dysautonomia, chronically elevated antihistone antibodies, and UC.

**Fig 1:**
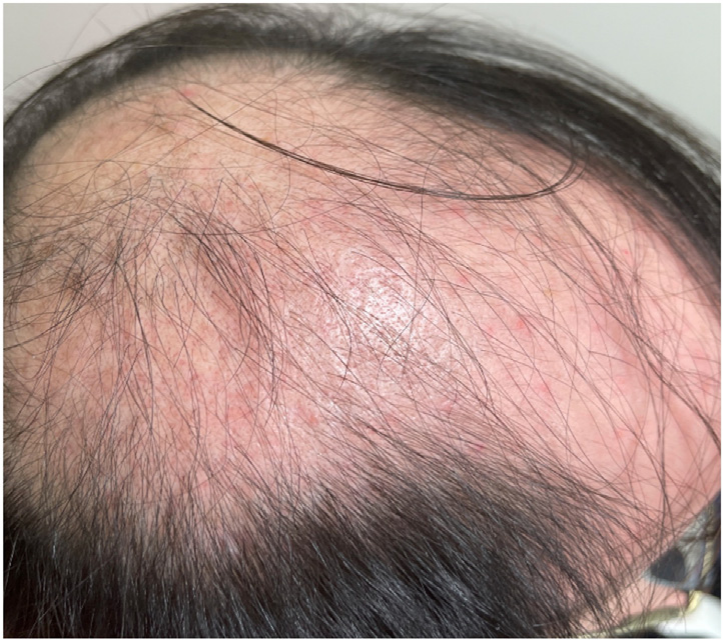
The vertex scalp had cicatricial alopecic patches; intact hair follicles demonstrated perifollicular scale and erythema.

**Fig 2:**
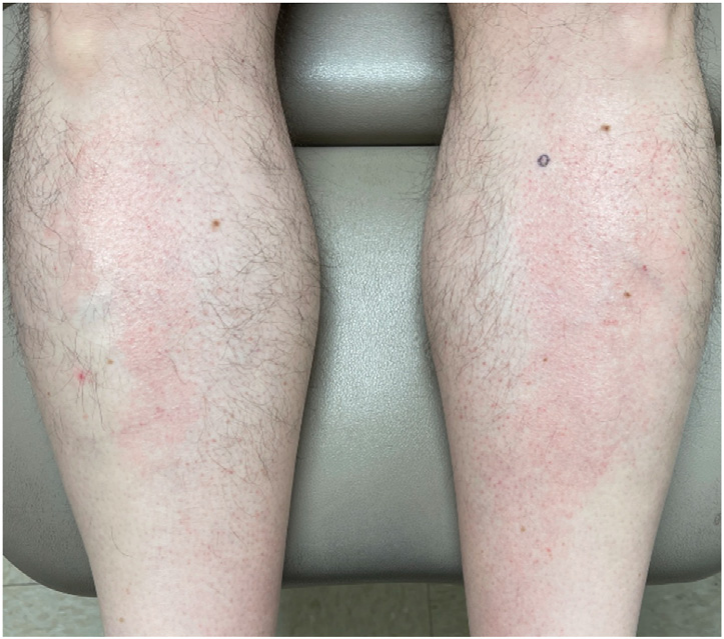
Erythematous, alopecic patches with follicular-based spinous papules extended over the bilateral anterior shins.

**Fig 3:**
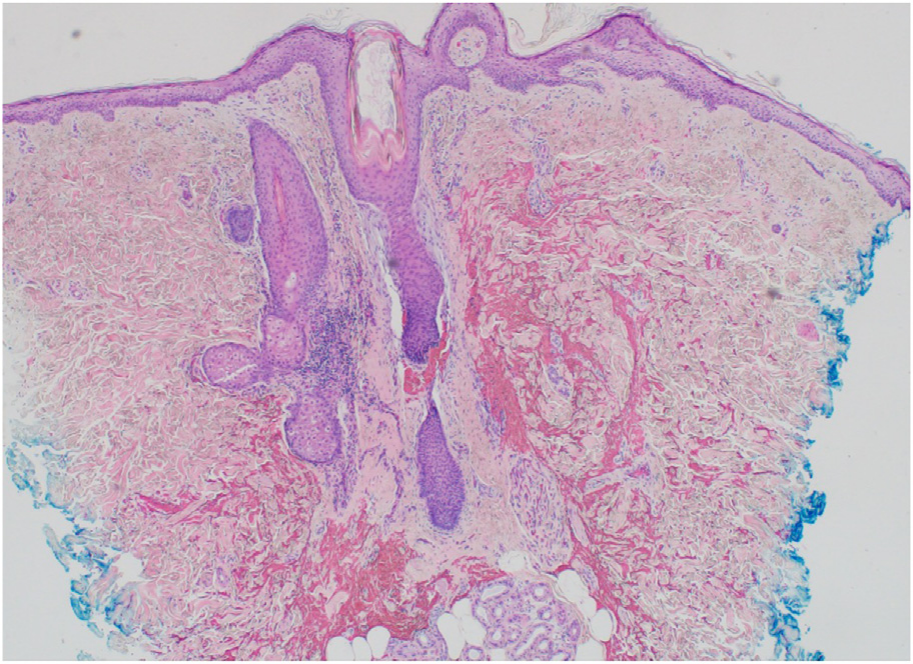
Low power punch biopsy from the lower leg shows 2 hair follicles with perifollicular lymphocytic inflammation, mucinous fibrosis, and hemorrhage (hematoxylin and eosin [H&E], 40×).

**Fig 4:**
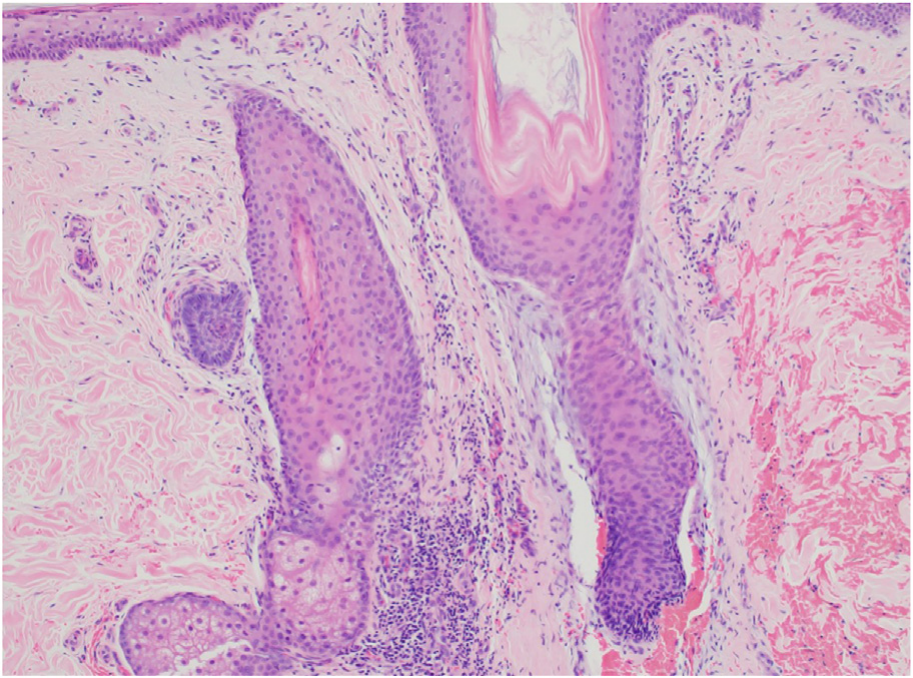
Medium power shows concentric perifollicular mucinous fibrosis of the isthmus portion of the follicle with epithelial thinning, lymphocytic inflammation, and perifollicular hemorrhage (H&E, 100×).

Physical examination of the scalp revealed alopecic patches with perifollicular erythema and scale (Fig 1). Chest and bilateral lower extremities demonstrated erythematous, alopecic macules and patches with follicular-based spinous papules (Fig 2). Biopsy was obtained and demonstrated focal perifollicular mucinous fibrosis and lymphocytic infiltrate with abundant red blood cell extravasation (Figs 3 and 4).

The patient was diagnosed with GLPLS. Treatment with topical corticosteroids and tacrolimus was ineffective. Prednisone, used to treat his other comorbidities, provided minimal relief of symptoms. Ultimately, hydroxychloroquine therapy was initiated.

## Discussion

GLPLS is a rare condition with approximately 50 reported cases in the literature.^4^ GLPLS typically presents in middle-aged, White females with fibrosing alopecia of the scalp, nonfibrosing alopecia of axillae and mons pubis, and scattered follicular spinous papules.^1^^,^^2^ While these findings can occur in any order, alopecia of the scalp frequently precedes the development of follicular spinous papules by months to years.^3^

GLPLS is thought to be T-cell-mediated with increased interferon-gamma dysregulation leading to increased antigen presentation to T-cells.^5^ This T-cell reaction may be triggered by medications, viruses, or contact sensitizers. Resulting lymphohistiocytic infiltration of the upper half of the pilosebaceous unit causes the destruction of basal stem cells of the bulge region.^5^

GLPLS has been associated with autoimmunity, hepatitis B virus vaccination, vitamin A deficiency, androgen insensitivity, and genetic disposition; associations with UC and autoimmune dysautonomia have not been previously reported.^2^^,^^6^ Of interest, GLPLS and UC share similar pathophysiologic pathways as both are mediated by interferon-gamma and T-cell activation.^7^ In contrast, autoimmune dysautonomia is attributed to antibody-mediated immunologic dysfunction.^8^ While antibodies are not strongly implicated in the etiology of GLPLS, one report identified autoantibodies to a centromere protein which plays a key role in chromosomal segregation and mitosis regulation.^6^ Acute sun damage as implicated in this case has not been previously reported to trigger GLPLS. However, LPP occurring in the setting of chronic actinic damage has been described.^9^

GLPLS treatment options are extrapolated from lichen planus and LPP management.^3^^,^^4^ This patient experienced limited benefit from oral corticosteroids with temporary improvement in pruritis. Treatment with hydroxychloroquine was initiated, but the patient was lost to follow-up. The immunosuppressive function of hydroxychloroquine is thought to result from its accumulation in lysosomes and inhibition of autophagosome fusion with lysosomes.^10^ This process inhibits antigen presentation, preventing T-cell activation, and may explain its demonstrated efficacy in GLPLS treatment.^10^ Early recognition and treatment of GLPLS can halt the progression of further hair loss, but full resolution of alopecia is not expected.

We report a unique case of GLPLS in a male patient with long-standing LPP who presented with fibrosing alopecia of nonscalp skin and sparing of typically involved axillary and pubic hair. This patient’s autoimmune dysautonomia and UC contribute to the spectrum of autoimmune conditions described with GLPLS. Notably, antecedent severe sunburn has not been previously reported. This case highlights the clinical variability of GLPLS, which may lead to challenges in diagnosis.

## Conflicts of interest

None disclosed.
